# Supplementary material for: Pregnane X receptor agonist nomilin extends lifespan and healthspan in preclinical models through detoxification functions
Source: Nat Commun. 2023 Jun 8;14:3368. doi: 10.1038/s41467-023-39118-9 (PMC10250385; doi:10.1038/s41467-023-39118-9)
Supplement: Supplementary file 1 — Supplementary Information [file 41467_2023_39118_MOESM1_ESM.pdf]

Supplementary Information for

Pregnane X receptor agonist nomilin extends lifespan and healthspan in preclinical models through detoxification functions

Shengjie Fan[1#], Yingxuan Yan[1#], Ying Xia[2,3#], Zhenyu Zhou[1#], Lingling Lou[1#], Mengnan Zhu[4,6#], Yongli Han[1], Deqiang Yao[5], Lijun Zhang[1], Minglv Fang[1], Lina Peng[4,6], Jing Yu[1], Ying Liu[1], Xiaoyan Gao[1], Huida Guan[7], Hongli Li[1], Changhong Wang[7], Xiaojun Wu[7], Huanhu Zhu[4]*, Yu Cao[2,3] *, Cheng Huang[1]*

Affiliations:

[1] School of Pharmacy, Shanghai University of Traditional Chinese Medicine, Shanghai, 201203, China.

[2] Department of Orthopaedics, Shanghai Key Laboratory of Orthopaedic Implant, Shanghai Ninth People's Hospital, Shanghai Jiao Tong University School of Medicine, Shanghai, 200011, China.

[3] Institute of Precision Medicine, the Ninth People’s Hospital, Shanghai Jiao Tong University School of Medicine, 115 Jinzun Road, Shanghai, 200125, China.

[4] School of Life Science and Technology, ShanghaiTech University, Shanghai 20121, China

[5] CAS Center for Excellence in Molecular Cell Science; Shanghai Institute of Biochemistry and Cell Biology, Chinese Academy of Sciences, Shanghai; University of Chinese Academy of Sciences, Beijing, 100049, China.

[6] iHuman Institute, ShanghaiTech University, Shanghai, 201210, China.

[7]Institute of Chinese Materia Medica, Shanghai University of Traditional Chinese Medicine, Shanghai, 201203, China.

This PDF file includes:

- Supplementary Figures 1 to 9
- Supplementary Tables 1 to 8
- Supplementary Methods (including Supplementary Results, Fig. S10 & Table S9)

Supplementary Fig. S1

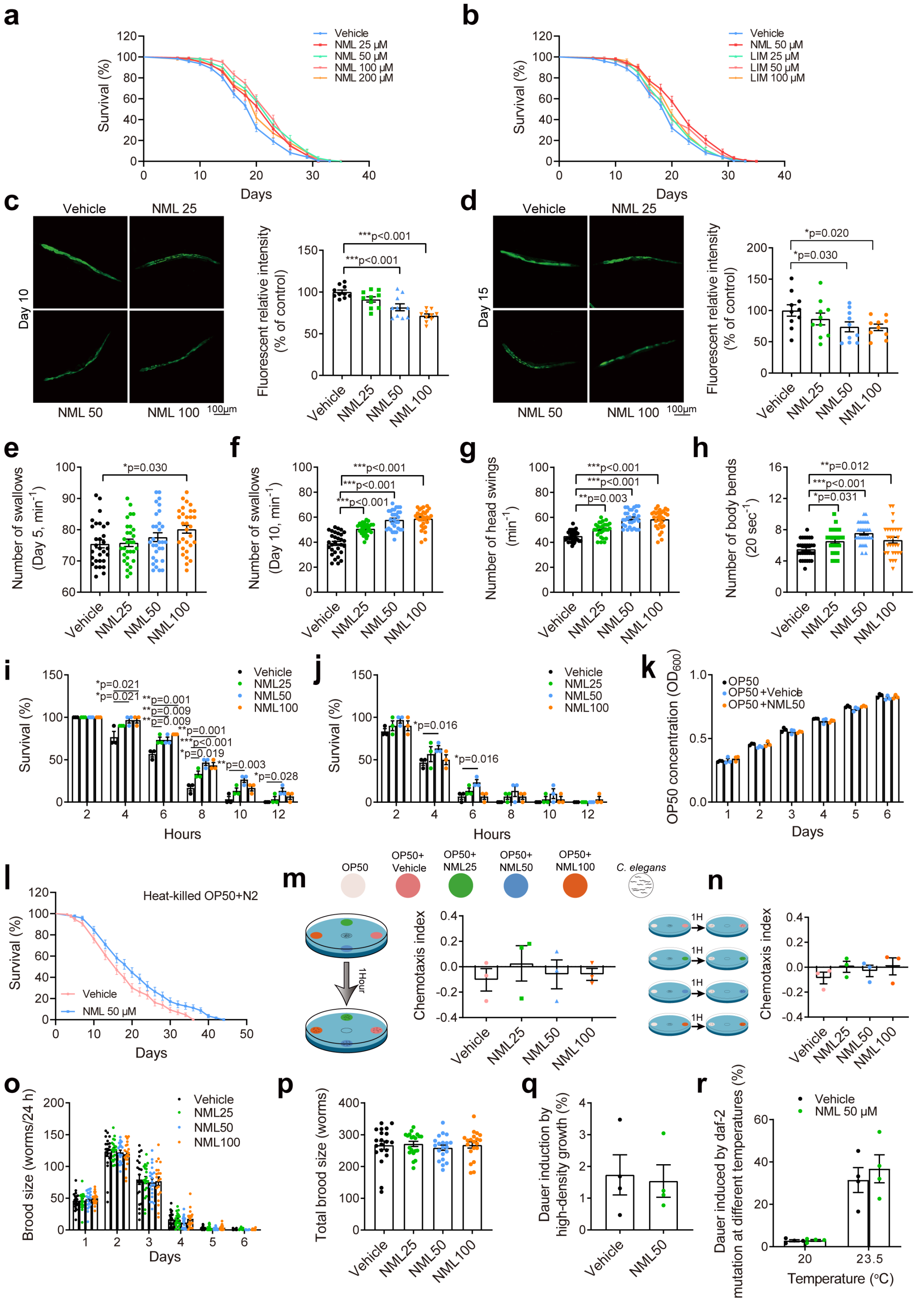

Fig. S1. Nomilin improves health-span and stress resistance in *C. elegans* without influence of food intake behavior and brood size. **a** Effects of different concentrations of nomilin on the lifespan of *C. elegans*. **b** Comparison of the effects between nomilin and limonin on the lifespan of *C. elegans*. **c-d**, Effects of nomilin on

the lipofuscin in day 10 (**c**) and day 15 (**d)** adult N2 *C. elegans*. The fluorescent relative intensity of lipofuscin in day 10 and 15 old adult worms (**c**, **d** n = 10/each). **e-f**, Effect of NML on the swallowing ability of day 5 and 10 N2 *C. elegans* (n = 30/each). **g**, Effects of nomilin on the head swings of N2 *C. elegans*. **h**, Effects of NML on the body bends of N2 *C. elegans*. (**g-h**, n = 30/each). **i**, Effects of nomilin on the survival percent of N2 worms on heat stress (35^0C). **j**, Effects of nomilin on the survival percent of N2 worms under oxidative stress (0.5‰ H_2O_2). The worms were treated with nomilin from L4 to day 10 (**i-j**, n = 3/each, each contains 50 worms). **k**, Effect of nomilin on the OP50 bacterial growth (n = 3/each). **l**, Lifespan curves of nomilin on N2 *C. elegans* fed with heat-killed OP50. **m-n**, Chemotaxis index of N2 *C. elegans* to DMSO, 25, 50, and 100 μM of nomilin *vs*. OP50. **o-p**: Effects of nomilin on the fertility of N2 *C. elegans*. Brood size (worms/24 h) (**o**), and total brood size (**p**). n = 60 (**k, m**); and 20 (**m, n**) worms per group. **q**, **r** Bar graphs showing the percentage of dauers of WT (**q**) or temperature sensitive Daf-c mutant *daf-2(e1370)* (**r**) under indicated conditions. Dietary supplementation of 50 μM nomilin did not enhance the dauer formation in all conditions. n = 4. Significance was calculated by (**c-h, m, n, p, q**) one-way ANOVA; (**i-k, r**) two-way ANOVA. All data were expressed as mean $\pm$ SEM. $^*p < 0.05$, $^{**}p < 0.01$, $^{***}p < 0.001$ *vs*. control group. Detailed information of (**a**, **b**, **l**) is shown in Supplementary Table S2.

Supplementary Fig. S2

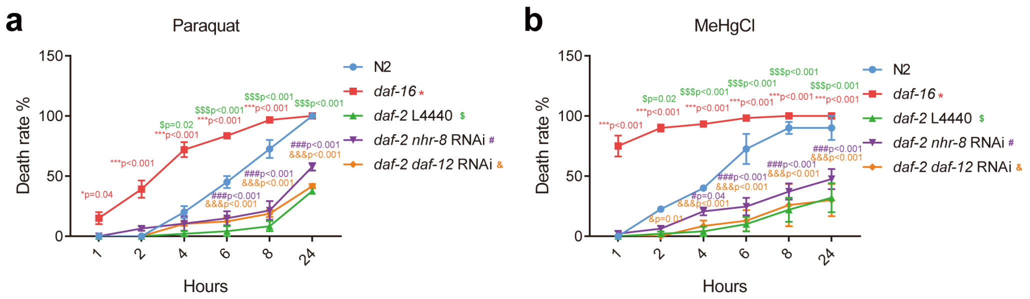

Fig. S2. IIS signaling is involved in detoxification functions of *nhr-8* and *daf-12*. Death rate curve of N2, *daf-16*, *daf-2* and *daf-2* with *nhr-8* or *daf-12* RNAi (daf-2::nhr-8, daf-2::daf-12) under paraquat (200 mM) (**a**) and MeHgCl (2 μM) (**b**) challenging. *n*=8 for N2, *daf-2* with *nhr-8* RNAi and *daf-2* with *daf-12* RNAi, *n*=12 for *daf-16*. All data were expressed as mean ± SEM. *p* values were determined by two-way ANOVA test. $^{***}p < 0.001$: *daf-16 vs*. control group; $^{\$\$\$}p < 0.001$: *daf-2 vs*. control group; $^{\#\#\#}p < 0.001$: *daf-2* with *nhr-8* RNAi *vs*. control group; $^{\&\&\&}p < 0.001$: *daf-2* with *daf-12* RNAi *vs*. control group.

Supplementary Fig. S3

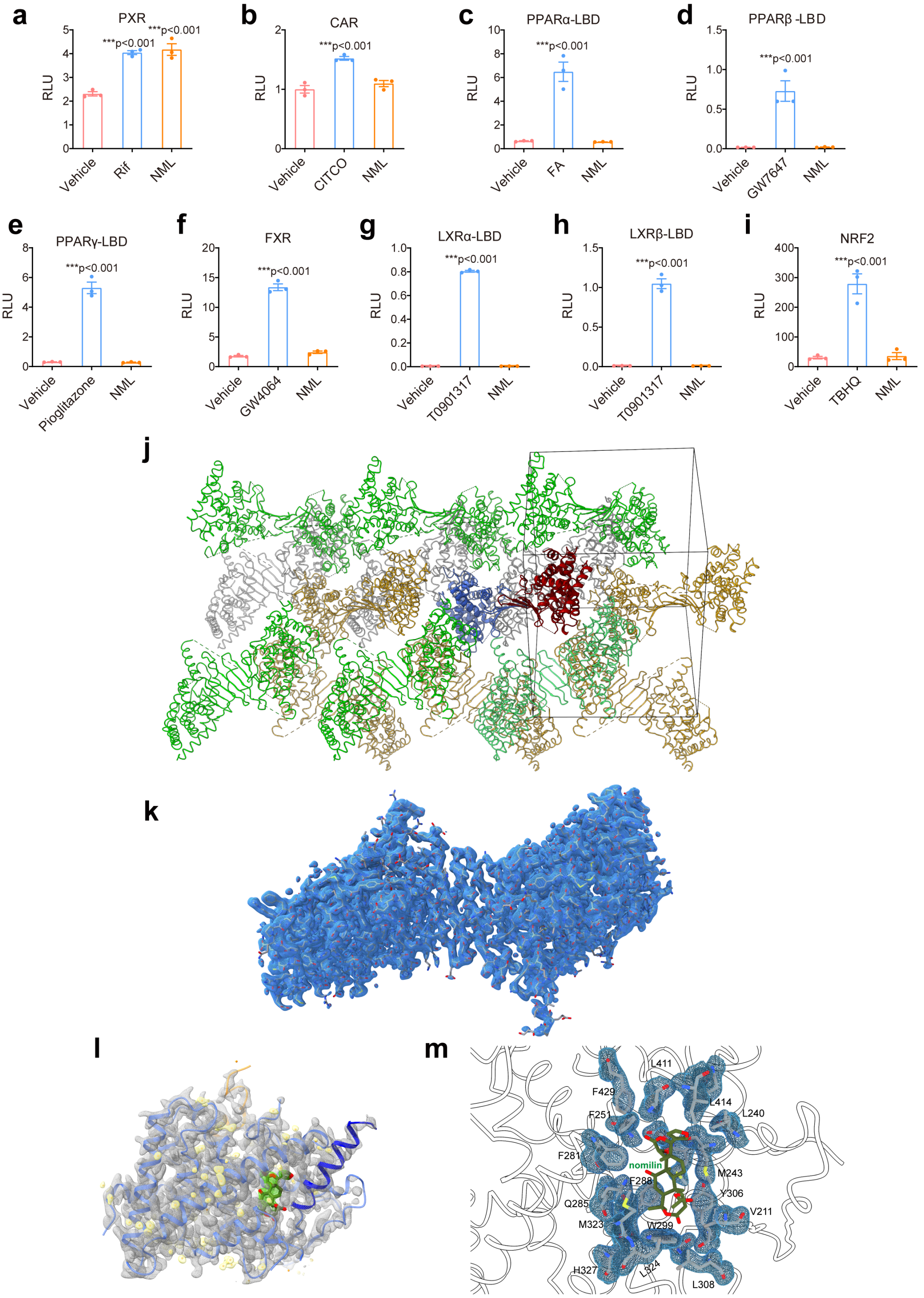

Fig. S3. Nomilin acts on PXR specifically. Reporter gene assay of hPXR (**a**), hCAR (**b**), PPARα (**c**), PPARβ (**d**), PPARγ (**e**), FXR (**f**), LXRα (**g**), LXRβ (**h**) and Nrf2-LBD (**i**). Rif (Rifampicin), CITCO (6-(4-chlorophenyl)imidazo[2,1-b] [1,3]

thiazole-5-carbaldehyde O-(3,4-dichlorobenzyl)oxime), FA (fenofibrate), GW7647, pioglitazone, GW4064, T0901317 and tBHQ (tert-Butylhydroquinone) were used as agonist controls. The plasmids were transfected to HEK293T cells and treated with nomilin or agonists at 10 μM for 24 h. The relative luciferase activities were measured by comparison to *Renilla* luciferase activities. *p* values were determined by one-way ANOVA test (**a-i**), $n = 3$/each. The data were shown as mean ± SEM. ***$p < 0.001$ compared to the control. CAR: constitutive androstane receptor; FXR: farnesoid X receptor; LXR: liver X receptor; NRF2: nuclear factor-erythroid 2-related factor 2; PPAR: peroxisome proliferators-activated receptor. **j** Crystal packing of human PXR^{LBD}-$NCOA1^{676\text{-}700}$. **k** Experimental electron density map of dimeric $hPXR^{LBD}$-$NCOA1^{676\text{-}700}$. The 2Fo-Fc electron density was shown as blue mesh and contoured to 1.5 σ. **l** Experimental electron density map of monomeric $hPXR^{LBD}$-$NCOA1^{676\text{-}700}$. The 2Fo-Fc electron density was shown as gray mesh for $hPXR^{LBD}$-$NCOA1^{676\text{-}700}$ protein, green mesh for nomilin molecule, and yellow solid surface for water molecules. The electron density maps were contoured to 1.0 σ for the nomilin and 1.2 σ for the protein and water. The protein was shown as cartoon model and colored in dark blue for the helix193-209, organe for the co-activator peptide, and light blue for the rest part. The water molecule was shown as yellow spheres and the nomilin as stick model colored by elements. **m** The electron density corresponding to the residues surrounding nomilin in protomer. The 2Fo-Fc electron density was shown as blue mesh and contoured to 1.5 σ.

Supplementary Fig. S4

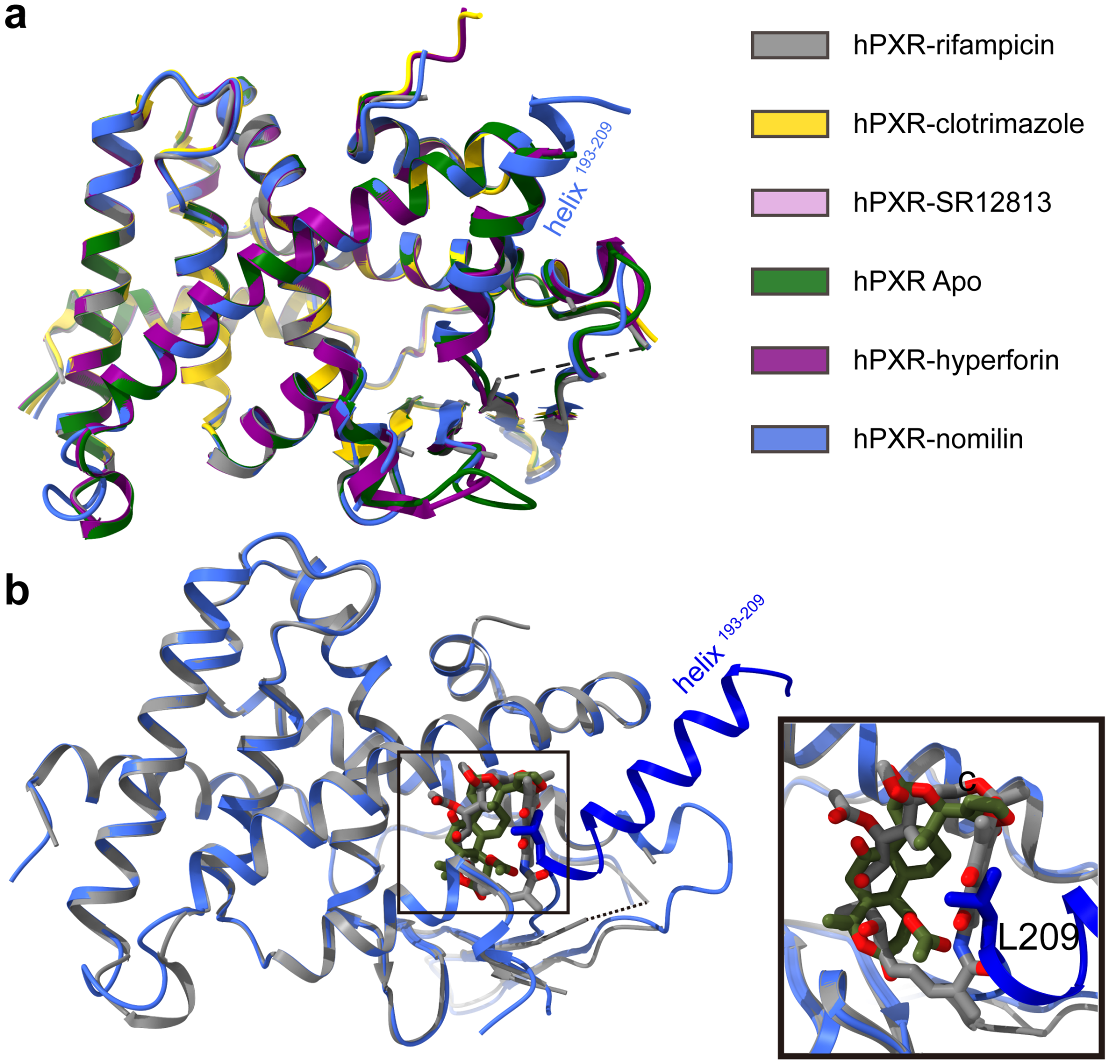

Fig. S4. Structural comparison among the human PXR in complex with compounds. **a** Structural superposition among hPXRs bound with nomilin, rifampicin (PDB ID 1SKX), clotrimazole (PDB ID 7AXA), SR12813 (PDB ID 1NRL), and hyperforin (PDB ID 1M13), as well as in unbound state (PDB ID 1ILG). The dotted line shows the unsolved helix$^{193\text{-}209}$ in hPXR-rifampcin structure. **b** The spatial clash between the helix$^{193\text{-}209}$ in hPXR-nomilin structrue and the rifampicin. The structural models for hPXR-nomilin and hPXR-rifampicin were superposed and shown as cartoon model. The helix comprising with amino acid 193-209 was highlighted in dark blue in hPXR-nomilin stucture and the corresponding part unsolved in hPXR-rifampicin structure was indicated with dot line. The nomilin and rifampicin were shown as stick models and color in green-red and gray-red, respectively. The binding pocket was enlarged to show the details in the right frame, with L209 shown as stick and colored in blue.

Supplementary Fig. S5

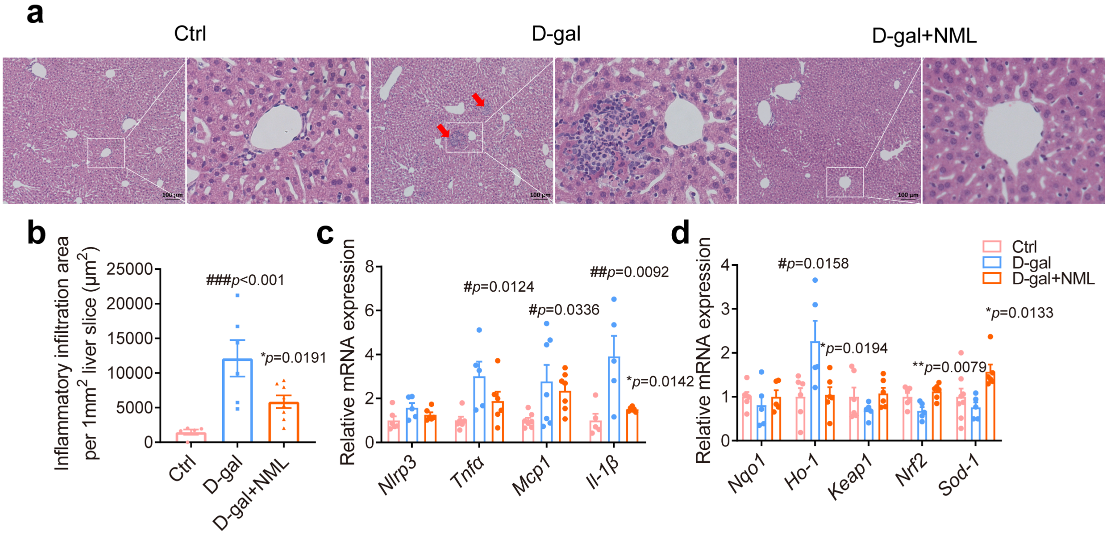

Fig. S5. Effects of nomilin on the liver damage of mice induced by D-galactose. **a** H&E staining of liver sections in D-galactose-treated mice. Red arrow: inflammatory filtration. **b** Inflammatory infiltration area per mm^2 liver slice (n=5 for Ctrl, n=6 for D-gal, n=8 for D-gal+NML). The expression of Inflammation related genes (**c**, n=5-7 for Ctrl, n=5-7 for D-gal, n=6-7 for D-gal+NML) and anti-oxidation genes (**d**, n=6-8 for Ctrl, n=5-6 for D-gal and D-gal+NML) determined by quantitative real time PCR. *β-Actin* was used as an internal control. The data were shown as mean ± SEM. *p* values were determined by one-way ANOVA test. ###*p*<0.001 *vs* the control group; ****p*<0.001 *vs* the D-galactose group.

Supplementary Fig. S6

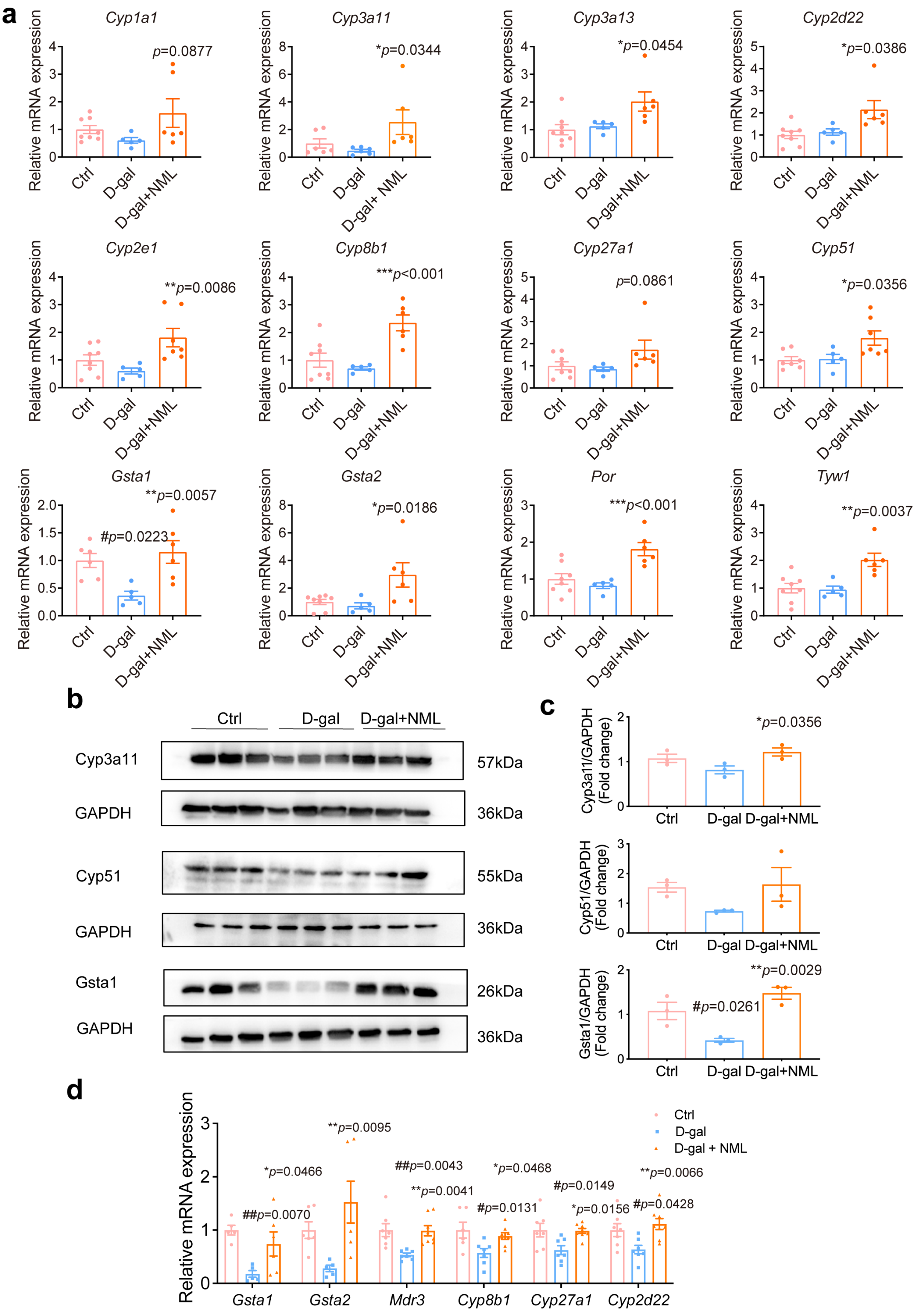

Fig. S6. Effects of nomilin on the expression of PXR downstream genes in the liver and the brain of D-galactose-treated mice. a The expression of PXR target genes in the liver of D-galactose-treated mice (*n*=6-8 for Ctrl, n=5-6 for D-gal, n=6-7 for D-gal+NML). *β-Actin* was used as an internal control. **b** Western blotting analysis of Cyp3a11, Cyp51a1, and Gsta1. GAPDH was used as loading control. **c**

Quantification of protein levels in (**b**). (*n* =3/group). **d** The expression of PXR target genes in the brain of D-galactose-treated mice (*n*=6-7 for Ctrl, *n*=5-7 for D-gal, *n*=6-8 for D-gal+NML). *β-Actin* was used as an internal control. The data were shown as mean ± SEM. *p* values were determined by one-way ANOVA test. ***$p < 0.001$ *vs* the D-galactose group.

Supplementary Fig. S7

a

Pairwise Comparisons

	group	DOX Chi-Square	DOX Sig.	NML Chi-Square	NML Sig.
Log Rank (Mantel-Cox)	DOX			5.312	.021
	NML	5.312	.021		

b

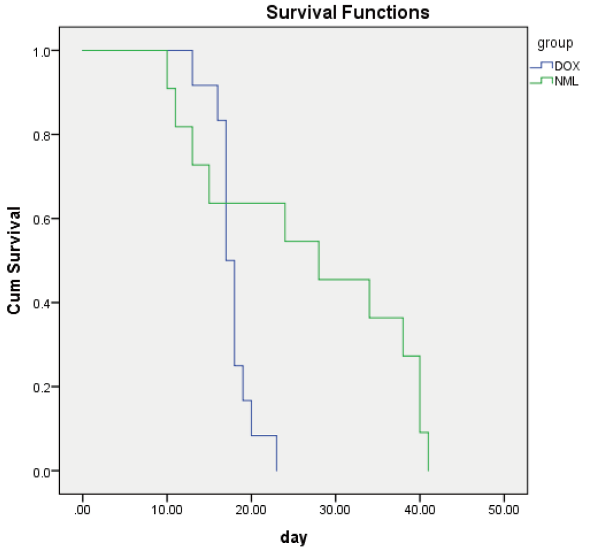

Fig. S7. Log-rank test (a) and Kaplan-meier curve (b) for lifespan of doxorubicin-induced model mice.

Supplementary Fig. S8

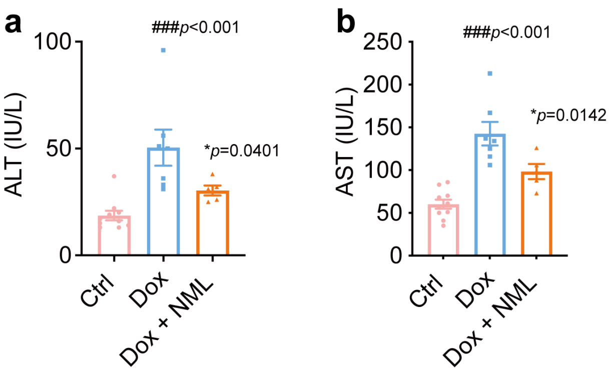

Fig. S8. Effects of nomilin on the liver function of mice treated by doxorubicin. The levels of serum alanine transaminase (ALT) (**a,** *n*=8 for Ctrl, *n*=7 for Dox, *n*=5 for Dox+NML) and aspartate aminotransferase (AST) (**b,** *n*=8 for Ctrl, *n*=7 for Dox, *n*=5 for Dox+NML). The data were shown as mean ± SEM. *p* values were determined by one-way ANOVA test. ### $p < 0.001$ *vs* Control group.

Supplementary Fig. S9

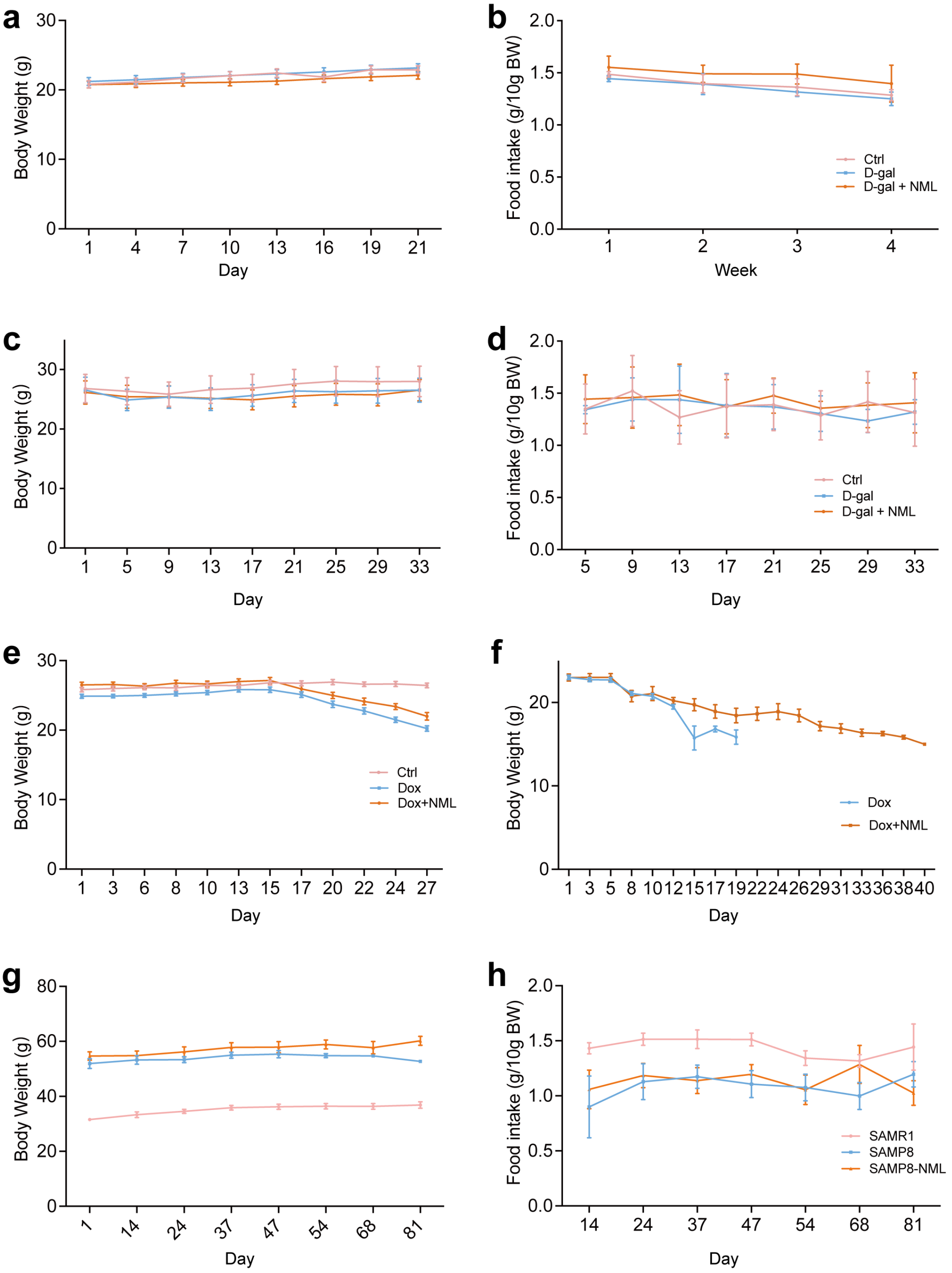

Fig. S9. Body weight and food intake. Body weight (**a**) and food intake (**b**) of D-galactose induced aging mice (n=20/group). Body weight (**c**) and food intake (**d**) of D-galactose treated PXR knockout mice (n=8/group). **e** Body weight of doxorubicin induced senescence mice in healthspan test (n=12/group). **f** Body weight of doxorubicin induced senescence mice in lifespan test (n=12 for DOX, n=11 for Dox+NML). Body weight (**g**) and food intake (**h**) of SAMP8 mice (n=9 for SAMR1 and SAMP8, n=10 for SAMP8-NML). The data were shown as mean ± SEM.

Supplementary Table S1. Effects of nomilin on the lifespan of N2, *daf-2, daf-16 and sir2.1 C. elegans*

Strains	Group	N	Mean life (days)	Maximum longevity (days)	Median survival time (days)	Censored subjects	Percent of prolong-life	p value
N2	Vehicle	172	16.30±0.34	25	15	9	-	-
	NML25	168	17.84±0.35*	29	17	12	9.43%	0.03
	NML50	166	20.28±0.42***	34	19	14	24.38%	<0.001
	NML100	159	20.16±0.42***	33	19	19	23.65%	<0.001
daf-2	Vehicle	218	43.16±0.69	62	44	7	-	
(e1370)	NML25	205	42.57±0.67$^{n.s.}$	63	43	15	-1.37%	>0.99
	NML50	225	44.44±0.60$^{n.s.}$	64	45	12	2.99%	0.88
	NML100	215	43.79±0.68$^{n.s.}$	64	45	21	1.46%	>0.99
daf-16	Vehicle	217	12.44±0.24	23	12	0	-	
(mu86)	NML25	224	12.23±0.25$^{n.s.}$	23	12	0	-1.66%	>0.99
	NML50	238	12.50±0.22$^{n.s.}$	24	12	0	0.46%	>0.99
	NML100	226	12.81±0.23$^{n.s.}$	24	12	0	2.98%	>0.99
Sir2.1	Vehicle	192	16.90±0.28	26	17	34	-	
(ok434)	NML25	202	17.90±0.26$^{n.s.}$	29	18	22	5.91%	0.07
	NML50	210	18.00±0.29*	29	18	23	6.51%	0.02
	NML100	202	17.90±0.29*	29	18	24	6.06%	0.05
raga-1	Vehicle	178	19.20±0.34	31	19	2	-	
(ok386)	NML25	167	20.52±0.39*	33	21	11	6.87%	0.05
	NML50	172	21.62±0.40***	35	21	5	12.57%	<0.001
	NML100	167	20.78±0.43*	33	21	9	8.24%	0.01

Notes: Lifespan experiments were analysed using Kaplan-Meier survival analysis and compared among groups, scoring for significance using the log-rank test. All data were expressed as mean± SEM. *n*: Sample numbers in each group. n.s., no significance. **p* < 0.05, ***p* < 0.01, ****p* < 0.001 *vs.* control group. NML, nomilin.

Supplementary Table S2. Effects of nomilin, limonin and heat-killed OP50 on the lifespan of *C. elegans*

Strains	Group	N	Mean life (days)	Maximum longevity (days)	Median survival time (days)	Censored subjects	Percent of prolong-life	p value
N2	Vehicle	141	19.20 ± 0.50	20	33	27	-	-
	NML25	106	21.26 ± 0.56**	23	31	82	10.73	0.006
	NML50	101	22.18 ± 0.58***	23	35	94	15.52	< 0.001
	NML100	118	22.54 ± 0.46***	23	35	81	17.40	< 0.001
	NML200	108	20.99 ± 0.57**	20	33	79	9.32	0.009
N2	Vehicle	141	19.20 ± 0.50	20	33	27	-	-
	LIM25	139	20.34 ± 0.46$^{n.s.}$	20	33	64	5.94	0.112
	LIM50	115	20.78 ± 0.55*	20	33	72	8.23	0.023
	LIM100	107	20.91 ± 0.50*	20	33	82	8.91	0.036
N2 fed with heat-killed OP50	Vehicle	130	18.01±0.73	36	16	66	-	-
	NML50	117	21.342±0.91**	44	20	69	18.47%	0.002

Notes: Lifespan experiments were analysed using Kaplan-Meier survival analysis and compared among groups, scoring for significance using the log-rank test. All data were expressed as mean± SEM. *n*: Sample numbers in each group. **p* < 0.05, ***p* < 0.01, ****p* < 0.001 *vs.* control group. n.s., no significance. NML, nomilin. LIM: limonin.

Supplementary Table S3. Summary of the effects of NML on the lifespans of *nhr-8* and *daf-12* mutants

Strains	Group	n	Mean life (days)	Maximum longevity (days)	Median survival time (days)	Censored subjects	Percent of prolong-life	p value
N2	Vehicle	174	21.14±0.45	34	22	15	-	
	NML50	165	25.12±0.47***	40	26	17	18.81%	<0.001
nhr-8 (tm1800)	Vehicle	130	21.46±0.59	38	22	48	-	
	NML50	191	21.41±0.43$^{n.s.}$	38	22	2	-0.22%	0.81
daf-12 (AA86)	Vehicle	167	13.57±0.36	28	12	18	-	
	NML50	158	13.27±0.36$^{n.s.}$	26	12	28	-2.23%	0.72

Note: Lifespan experiments were analysed using Kaplan-Meier survival analysis and compared among groups, scoring for significance using the log-rank test. All data were expressed as mean ± SEM. *n*: Sample numbers in each group. n.s., no significance. *** $p < 0.001$ *vs*. control group. NML, nomilin.

Supplementary Table S4. Effects of nomilin on the lifespan of detoxification gene RNAi *C. elegans*

Strains and RNAi	Group	N	Mean life (days)	Maximum longevity (days)	Median survival time (days)	Censored subjects	Percent of prolong-life	p value
L4440	Vehicle	183	20.67 ± 0.52	21	36	29	-	
	NML50	122	25.40 ± 0.69***	24	36	80	22.88%	<0.001
cyp35a3	Vehicle	148	21.07 ± 0.51	24	32	48	-	
	NML50	166	19.35 ± 0.51$^{n.s.}$	21	38	34	-0.08%	0.069
gst-4	Vehicle	182	25.55 ± 0.53	27	38	17	-	
	NML50	174	25.60 ± 0.47$^{n.s.}$	27	39	17	-0.00%	0.322
pgp-3	Vehicle	131	24.41 ± 0.59	27	38	43	-	
	NML50	126	23.33 ± 0.55$^{n.s.}$	27	39	71	-0.04%	0.101
pgp-14	Vehicle	158	20.31 ± 0.56	21	35	24	-	
	NML50	147	24.52 ± 0.50***	24	38	23	20.73%	<0.001

Notes: Lifespan experiments were analysed using Kaplan-Meier survival analysis and compared among groups, scoring for significance using the log-rank test. All data were expressed as mean± SEM. *n*: Sample numbers in each group. n.s., no significance. *$p < 0.05$, **$p < 0.01$, ***$p < 0.001$ *vs.* control group. NML, nomilin.

Supplementary Table S5. Statistics for the highest-resolution shell are shown in parentheses

	**Human PXR^{LBD}-$NCOA1^{676-700}$**
**Wavelength (Å)**	0.97890
**Resolution range (Å)**	37.3 - 2.103 (2.178 - 2.103)
**Space group**	$P\ 2_1\ 2_1\ 2_1$
**a, b, c (Å)**	84.986, 90.163, 106.391
**α, β, γ (°)**	90.0, 90.0, 90.0
**Total reflections**	318066 (31505)
**Unique reflections**	48171 (4750)
**Multiplicity**	6.60
**Completeness (%)**	99.00
**I/sigma**	19.68 (2.30)
**Wilson B-factor (Å ^{2})**	41.73
**R_{merge}**	0.05394 (0.7082)
**R_{meas}**	0.0587 (0.7685)
**R_{pim}**	0.02288 (0.2957)
**$CC_{1/2}$**	0.999 (0.809)
**Reflections used in refinement**	47751 (4749)
**Reflections used for R-free**	1996 (191)
**R_{work}**	0.1957 (0.2593)
**R_{free}**	0.2274 (0.3119)
**CC_{work}**	0.963 (0.859)
**CC_{free}**	0.944 (0.805)
**Number of non-hydrogen atoms**	4981
**macromolecules**	4720
**ligands**	74
**solvent**	187
**Protein residues**	575
**RMS(bonds)**	0.008
**RMS(angles)**	1.00
**Ramachandran favored (%)**	96.24
**Ramachandran allowed (%)**	3.04
**Ramachandran outliers (%)**	0.72
**Rotamer outliers (%)**	0.19
**Clashscore**	6.60
**Average B-factor**	56.25
**macromolecules**	56.28
**ligands**	62.80
**solvent**	52.70

Statistics for the highest-resolution shell are shown in parentheses.

Supplementary Table S6. Summary of the effects of NML on the lifespans of *hPXR* transgenic N2, *nhr-8*, and *daf-12 C. elegans*

Strains	Group	n	Mean life (days)	Maximum longevity (days)	Median survival time (days)	Censored subjects	Percent of prolong-life	*p* value
N2-pSM	Vehicle	147	19.67±0.51	39	20	39	-	
	NML50	152	22.25±0.50***	39	22	40	13.10%	<0.001
N2-hPXR	Vehicle	133	18.68±0.59	40	20	49	-	
	NML50	155	22.61±0.54***	40	22	29	20.99%	<0.001
N2-hPXR^{S247R}	Vehicle	165	20.90±0.60	40	20	23	-	
	NML50	172	22.72±0.57*	41	22	24	8.70%	0.04
nhr-8-pSM	Vehicle	119	15.38±0.59	34	14	46	-	
	NML50	127	16.81±0.50*	34	16	41	9.32%	0.04
nhr-8-hPXR	Vehicle	118	17.51±0.60	34	16	16	-	
	NML50	115	19.96±0.64**	39	20	7	13.98%	0.005
nhr-8-hPXR^{S247R}	Vehicle	135	17.50±0.53	34	20	41	-	
	NML50	141	18.63±0.53$^{n.s.}$	34	20	33	6.49%	0.17
daf-12-pSM	Vehicle	156	15.15±0.42	28	14	28	-	
	NML50	168	16.13±0.41$^{n.s.}$	28	16	14	6.41%	0.11
daf-12-hPXR	Vehicle	148	16.58±0.51	39	16	22	-	
	NML50	149	18.32±0.51*	39	20	11	10.46%	0.02
daf-12-hPXR^{S247R}	Vehicle	141	15.17±0.48	31	14	37	-	
	NML50	152	16.18±0.44$^{n.s.}$	31	16	22	6.64%	0.09

Note: Lifespan experiments were analysed using Kaplan-Meier survival analysis and compared among groups, scoring for significance using the log-rank test. All data were expressed as mean ± SEM. *n*: Sample numbers in each group. n.s., no significance. **p* < 0.05, ***p* < 0.01, ****p* < 0.001 *vs*. control group. NML, nomilin.

Supplementary Table S7. Sequences of the primers for quantitative real-time PCR in mice

Gene	Forward primer	Reverse primer
β-Actin	TGTCCACCTTCCAGCAGATGT	AGCTCAGTAACAGTCCGCCTAGA
Nrf2	GCCTCCAAAGGATGTCAATCA	GCCTCACCTCTGCTGCAAGTA
Nqo1	TGGCGTAGTTGAATGATGTCTT	TTCGGTATTACGATCCTCCCT
Ho-1	CCACATTGGACAGAGTTCACAG	CCTCACAGATGGCGTCACTTC
Tnf-α	ATGGATCTCAAAGACAACCAACTAG	ACGGCAGAGAGGAGGTTGACTT
IL-1β	TCGTGCTGTCGGACCCATAT	GGTTCTCCTTGTACAAAGCTCATG
Nlrp3	AGCCAGAGTGGAATGACACG	GCGCGTTCCTGTCCTTGATA
Mcp1	AGGTCCCTGTCATGCTTC	GTGCTTGAGGTGGTTGTG
p16INK4A	GCCGTGTGCATGACGTG	TTGCCCATCATCATCACCTGAA
Il-6	TTCCTCTGGTCTTCTGGAGT	TCTGTGACTCCAGCTTATCTCTTG
Cyp27a1	GCACAGGAGAGTACGGAGG	CGGGCAAGTGCAGCACATA
Cyp2d22	GAAGACCTTGCCAACCAG	CACCCTTTCAGCCCTAAC
Cyp3a13	GATTCTTGCTTACCAGAAGGGC	GCCGGTTTGTGAAGGTAGAGTA
Cyp8b1	GGACAGCCTATCCTTGGTGA	GACGGAACTTCCTGAACAGC
Por	TCCAGACTTCGCTTCATACTC	CTATTCCATTGCCTCGTCG
Tyw1	CAGTTGGAGTGCTCGTGTT	AGGAAGTGGTTCGGTTTG
Cyp51	CTGAAACTTGGCAGAGGC	CTCAACGAGAAGGTGGCT
Cyp2j6	ACATAACCTCGTCCAGTAA	ACCTTTCAACCCTCACTT
Cyp1a1	CAGAGCCAGTAACCTCCC	TTGGTCGTGTCAGTAGCC
Gsta1	CTTCCCTGCCTTTGAAAAAGTCT	TCTGGGCTGTGAAATGGGTC
Gsta2	GCAGGGGTGGAGTTTGAAGA	AGAATGGCTCTGGTCTGCAC
Cyp3a11	TACTTTTTCCATTCCTG	TCCTTCATTCTGTCCAC
Cyp2e1	CCAAGTCTTTAACCAAGTTGGCA	CCTTGACAGCCTTGTAGCCA
Mdr3	ATGGCCCTACTTTGTCGTGG	CTGCTTCACTGCATCATCGC

Supplementary Table S8. Sequences of the primers for quantitative real-time PCR in *C. elegans*

Gene	F primer	R primer
act-1	CGCCATCCTCCGTCTTGACTTG	GCTCAGCGGTGGTGGTGAAAG
daf-2	TGGGAGCTACGGCAGGATGATG	GCACACGGTCCGAAACGATCAC
daf-16	CGGATACCGTACTCGTGATGAT	CCAAACAGCCACCCAAATCA
sod-2	GCTCTTCAGCCAGCTCTC	AGTATCCCAACCATCCCC
sod-3	ATTAAGCGCGACTTCGGTTCCC	TCCCCAGCCAGAGCCTTGAAC
clk-1	ATACTGCTGCTTCTCGTC	TCATCCCACATCTTTTTG
clk-2	AGATGTGGCGTATGTCCT	CGTAGTTTTGCGTTTTCA
acs-19	CATCTTCTGTCTACTGCT	ATTGATTCTCTCTCCAAC
unc-84	CTCCTGAACCAACTTTTG	CTGTCGCTTCTTCGTATT
lin-2	AGGAGGGCAATTGTGTAT	CGTCGTATGATGTGTGGA
hsp-16.2	GGCTCTGATGGAACGCCAAT	TGAGACGTTGAGATTGATGGCA
pha-4	GAAGCCGAGCCAGCCACAAC	TCGGAGGTCCCAGAAGTGGTTC
cyp-13a7	AAAAATGGCAATGGGACAAG	AATACTTTGAATATCGGTAG
cyp-13a11	GCAAATTCTCGCCGTTGTAT	TCGTCTCCTGATTCCCATCT
cyp-14a1	CCTTTCTTGGGGTCTCATCA	AAGTAGCGGCTTGGATTGAA
cyp-14a3	CAGGCACTGGAGACAAATCA	GCAAAAGAGAATGGGGGATT
cyp-34a9	AGCAAGGCAGAAACTTCCAA	ACCTGTGCCCAAAAGTGTTC
cyp-35a1	CGGAGTCACTGTTGCTCAAGCC	AGACTTCAAACGCAGCACCCATG
cyp-35a2	ACTGGTGGCATTGTTTCGACTCTC	GGAATTGGTCCGACCCATAGTGTG
cyp-35a3	GCTCAACTCAGTGCTCTCCATGTC	TCCCAGGCAACTTCTCTTTCCAAC
cyp-35a4	CTGACCGTGCTTCAACTCCATACC	TCCAGCATCGACAGGGTGACC
cyp-35a5	GGGAAGGAGCCGATGGAAATCAAG	GGGAAGGAGCCGATGGAAATCAAG
cyp-35b1	TGAACACGAGATGTGCCGAA	AACGTTTTCCGACGAGCAGA
cyp-35b2	GTTCCTCCCGCCTGTTTTCT	TTTCCTCGCATCTTGCATCC
cyp-35b3	GTGATTATGAAACGTCGCAAGAAG	GCGGATGCTGTAAATGGAAAGAC
cyp-35C1	AAAGTGACTAACGGAGGATCTCG	CTAGCAAGAGCCGAGCTGTATTT
cyp-36a1	GGTGGAAGGCTCAACGACGATTC	GCCAACGAAGCAATTGTGTCCTG
cyp-37a1	AGATTTTCGAGGGGACAAGCA	ACTGATGGCCGCATTCTCAA
gst-4	TTTGATGCTCGTGCTCTTGC	CCAAATGGAGTCGTTGGCTTC
gst-10	GGAGTCCGCGATGTTCGTAT	TTCACTAGAGCCTCCGGGAT
ugt-44	GCACATTTTGGTATGCTCTGCT	CGGCAACAGAAGGGTCACAT

pgp-3	GTGATGGGACTTCCTGACGG	CTTTGGGTCTCTGACAATCGC
pgp-12	CCACTCATGTACCACGGCAT	AATAGCATTCCAGCGGCAGT
pgp-13	CCGATGGCATAGACACCGAA	GCTTCTTGCACAGCCCTTTC
pgp-14	AGGAGTACGGTGCTAGCGAT	ACATCTTTGGGGCGTCATCA
hpxr	GGACGCTCAGATGAAAACCT	AGCATGGGCTCCAGTAGAAG

Supplementary Methods

Sample preparation. The nomilin standard and nomilin and hPAR/nomilin crystal samples was diluted with HPLC grade methanol. After centrifugation at 13,000 g for 10 min, the supernatants were injected into the UHPLC-Q/TOF-MS system for analysis.

Instrument and analysis conditions. A UHPLC-Q/TOF-MS system, performed on a triple TOF^{TM} 5600^{+} system (AB Sciex, CA, USA) equipped with ESI mode and coupled to a Shimadzu 30A UHPLC system (Shimadzu, Kyoto, Japan) was used for the analysis of the sample. The mass spectrometer was operated in the positive mode with capillary voltages of 5.5 kV. The turbo spray temperature was 550 °C, and the declustering potential (DP) was 80 V. Nitrogen was used as the nebulizer and auxiliary gas, and the flow rates of nebulizer gas (gas 1), heater gas (gas 2), and curtain gas were set to 55, 55, and 35 L/min, respectively. For the full MS-IDA (information dependent acquisition analysis, the scan range was operated with mass *m/z* 50 to *m/z* 1200. The collision energy (CE) was set to 35 eV, and the collision energy spread (CES) was set to 15 eV. The analyte was separated using ACQUITY UPLC HSS T3 reverse phase column (2.1×100 mm, 1.8 μm). The mobile phase was a gradient system consisting of 0.1% formic acid in water (A) and with 0.1% formic acid in acetonitrile (B) with the gradient 5% B (0—1 min), 5%—65% B (1—3 min), 65%—95% B (3—10 min), 95% B (10—12 min). The column temperature was set to 45 °C, the flow rate was set to 0.4 mL/min, and the injection volume was 10 μL.

Supplementary Results

The total ion chromatograms and MS/MS fragmentation ions of reference substance nomilin and nomilin in crystallization system were showed in Figure 1. It could be seen from Figure 1a &b that the peaks (retention time was 4.22 min) were observed in the total ion chromatogram of reference substance nomilin in DMSO and nomilin in crystallization system, respectively. The analyte was calculated as $C_{28}H_{34}O_9$ based on the accurate mass measurement $[M+H]^+$ ion and MS/MS fragmentation ions (Figure 1c&d and Table 1), which was consistent with the reported references [1, 2]. The ion information of deacetylnomilin (formula was $C_{26}H_{32}O_8$) cannot be extracted from the mass spectrum. The results showed that the compound was exact nomilin, but not deacetylnomilin. It was speculated that the nomilin could not be modified basically during the crystallization process.

Supplementary Fig. S10

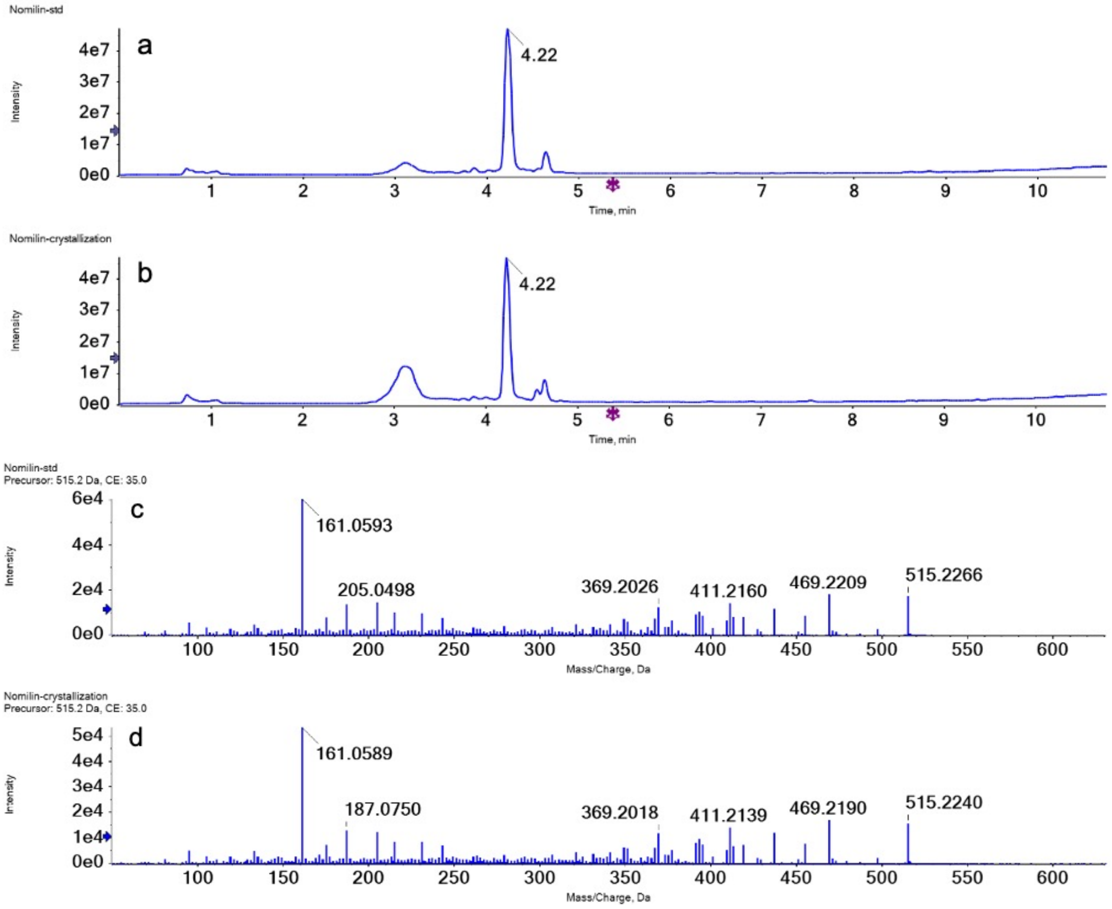

Fig. S10. Mass spectrometry analysis of nomilin in crystallization system. The total ion chromatogram of nomilinin reference substance solution (**a**) and in crystallization system (**b**). The MS/MS fragmentation ion chromatogram of nomilin in reference substance solution (**c**) and in crystallization system (**d**).

Supplementary Table S9. The identified results of high-resolution mass spectrometry of nomilin

Analytes	RT (min)	Formula	$[M+H]^+$			MS/MS Fragments	References
			m/z theory	*m/z* measured	Error (ppm)		
**Nomilin**	4.22	$C_{28}H_{34}O_9$	515.2276	515.2266	-1.9	469.2209, 411.2160, 369.2026, 161.0593	1,2
**Nomilin in crystallization system**	4.22	$C_{28}H_{34}O_9$	515.2276	515.2240	-6.9	469.2190, 411.2139, 369.2018, 161.0589	1,2

Supplementary References:

1 Goh RMV, Pua A, Ee KH, Huang Y, Liu SQ, Lassabliere B, Yu B. Investigation of changes in non-traditional indices of maturation in Navel orange peel and juice using GC-MS and LC-QTOF/MS. Food Res Int. 2021 Oct;148:110607. doi: 10.1016/j.foodres.2021.110607. Epub 2021 Jul 14. PMID: 34507751.

2 Avula B, Sagi S, Wang YH, Wang M, Gafner S, Manthey JA, Khan IA. Liquid Chromatography-Electrospray Ionization Mass Spectrometry Analysis of Limonoids and Flavonoids in Seeds of Grapefruits, Other Citrus Species, and Dietary Supplements. Planta Med. 2016 Jul;82(11-12):1058-69. doi: 10.1055/s-0042-107598. Epub 2016 May 25. PMID: 27224266.
